# Confronting allergies: strategies for combating pollution and safeguarding our health

**DOI:** 10.3389/falgy.2024.1521072

**Published:** 2025-01-07

**Authors:** Santanu Pattanayak, Suman Kalyan Dinda, Shreyasee Hazra, Rittwicka Mukhopadhyay, Samvabi Samanta, Shramalina Dey, Dipak Manna

**Affiliations:** ^1^ Department of Biomedical Science and Technology, School of Biological Sciences, Ramakrishna Mission Vivekananda Educational and Research Institute (RKMVERI), Kolkata, India; ^2^ Department of Agricultural Biotechnology, Ramakrishna Mission Vivekananda Educational and Research Institute (RKMVERI), Kolkata, India

**Keywords:** pollution, allergies, climate change, health problem, respiratory diseases, immunity, immunotherapy, antihistamines

## Abstract

Increasing evidence demonstrates a robust link between environmental pollutants and allergic reactions, with air and indoor pollution exacerbating respiratory allergies and climate change intensifying seasonal allergies. Comprehensive action, including government regulations, public awareness, and individual efforts, is essential to mitigate pollution's impact on allergies and safeguard public health and ecological balance. Recent findings indicate a strong correlation between environmental pollutants and allergic reactions, with air pollution from vehicular emissions and industrial activities exacerbating respiratory allergies like asthma and allergic rhinitis. Additionally, indoor pollutants such as mold and volatile organic compounds are significant triggers of allergic responses, especially among vulnerable populations. Furthermore, climate change, driven by pollution, is intensifying seasonal allergies due to altered weather patterns and increased pollen production. This review emphasizes the critical importance of addressing pollution and allergies, highlighting the growing concerns in contemporary society. This review highlights the urgent need to address pollution and allergies, emphasizing their increasing significance in modern society and outlining effective allergy management strategies.

## 1 Introduction

**Figure 1:**
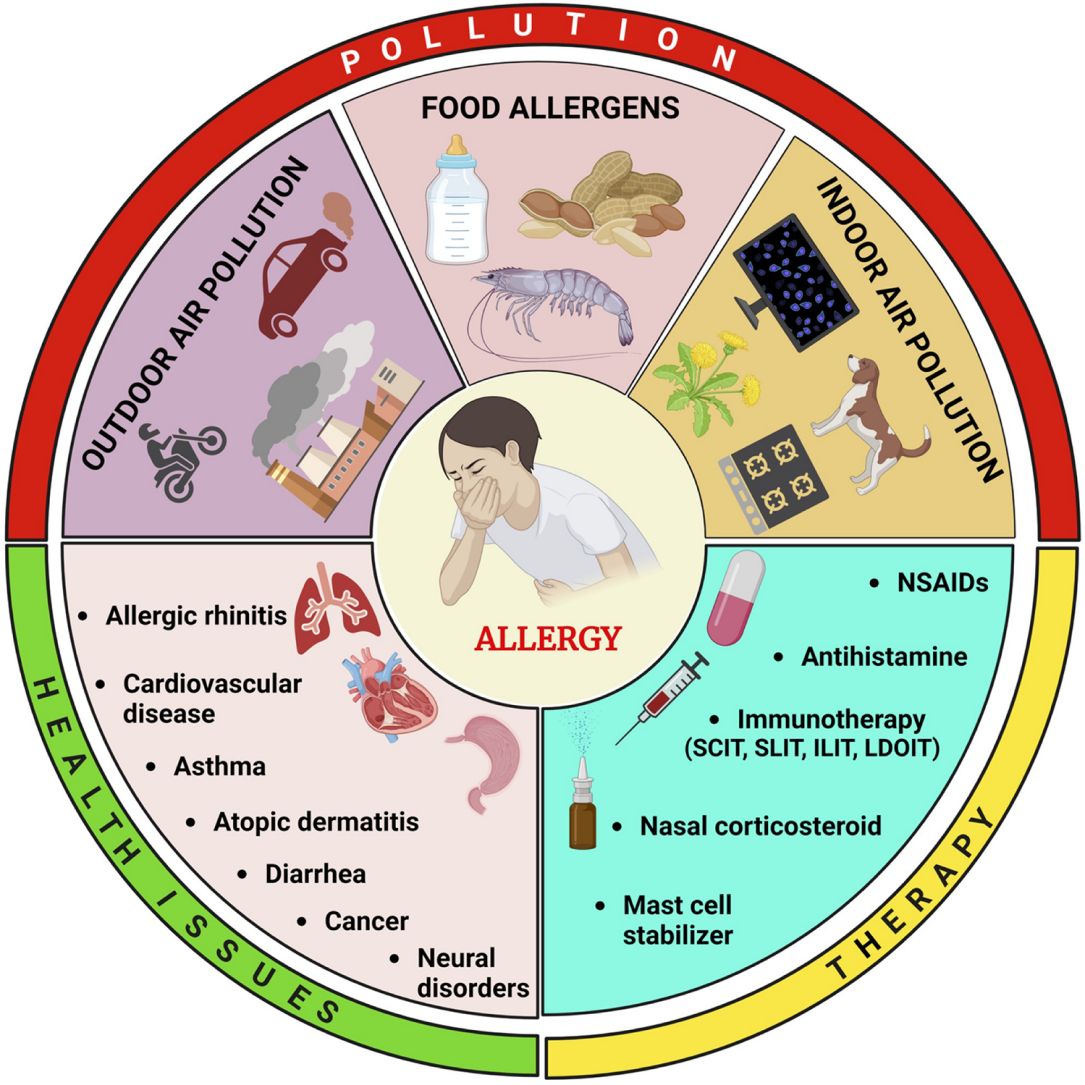
Illustration depicting the diverse origins of pollution and their repercussions on human health, alongside preventive approaches via therapeutic interventions. Allergens originating from outdoor and indoor air contamination as well as food sources can instigate various health complications including allergic rhinitis, cardiovascular diseases, asthma, atopic dermatitis, diarrhea, cancer, and neural disorders. These ailments can be mitigated through the utilization of non-steroidal anti-inflammatory drugs (NSAIDs), antihistamines, immunotherapies such as subcutaneous immunotherapy (SCIT), sublingual immunotherapy (SLIT), intra-lymphatic immunotherapy (ILIT), and low-dose oral immunotherapy (LDOIT), nasal corticosteroids, or the application of mast cell stabilizers.

Pollution and allergies have become pressing public health concerns with far-reaching implications worldwide. The growing body of evidence indicates a significant interplay between environmental pollutants and allergic reactions, prompting for exploring the intricate relationship between pollution and allergic conditions more comprehensively (1). Air pollution, resulting from vehicular emissions, industrial activities, and the burning of fossil fuels, is a major contributor to respiratory allergies such as asthma and allergic rhinitis (2). Particulate matter, nitrogen dioxide, sulfur dioxide, and ozone are among the most prominent air pollutants known to trigger allergic responses and compromise respiratory function. Long-term exposure to these pollutants has been promoted of an increased risk of developing allergies and exacerbating existing allergic conditions, burdening healthcare systems, and reducing quality of life for affected individuals (3). Apart from outdoor pollutants, indoor air quality also plays a critical role in triggering allergies. Indoor pollutants such as mold, pet dander, and volatile organic compounds (VOCs) can provoke allergic reactions, particularly in vulnerable populations like children and the elderly (4). Poor indoor air quality, especially in densely populated urban areas, can lead to a higher prevalence of allergies and respiratory illnesses, making it essential to address both indoor and outdoor pollution sources (Figure 1). Climate change, largely driven by escalating pollution levels, further complicates the allergy landscape. Altered weather patterns, increased temperatures, and extended growing seasons can affect plant growth and pollen production. As a result, seasonal allergies are becoming more severe and prolonged, causing discomfort and distress to individuals with allergies (5). Furthermore, rising carbon dioxide levels can enhance the allergenicity of plants, exacerbating the burden on allergic individuals and increasing the demand for medical resources. Pollution's impact extends beyond human health, significantly affecting wildlife and ecosystems (5). Pollutants can alter the behavior and reproductive success of various species, leading to ecological imbalances and unintended consequences on biodiversity (6). This interconnection between pollution, allergies, and ecological disturbances underscores the necessity of a holistic approach to address these challenges. Efforts to mitigate pollution's impact on allergies demand a multifaceted approach involving collaborative efforts from various stakeholders. Government regulations must be implemented to limit pollution levels, enforce emissions standards, and promote clean energy solutions (7). Public awareness campaigns play a crucial role in educating communities about the detrimental effects of pollution on allergic conditions and the importance of adopting eco-friendly practices (8). Individual actions are equally vital in curbing pollution and its impact on allergies. Reducing reliance on fossil fuels by embracing renewable energy sources, adopting sustainable transportation options, and promoting green practices in everyday life can significantly contribute to improving air quality and reducing allergen exposure (9). Improving indoor air quality is equally crucial. Proper ventilation, regular cleaning, and the use of air purifiers can help reduce exposure to indoor allergens, enhancing overall respiratory health and minimizing the risk of allergic reactions. In conclusion, the intertwining relationship between pollution and allergies presents a growing public health concern that warrants urgent attention and collective action. The adverse effects of environmental pollutants on human health, combined with the impact on wildlife and ecosystems, necessitate comprehensive and sustained efforts to combat pollution and its implications for allergic conditions (10). Recognizing the complexity of these challenges, it is imperative for governments, industries, communities, and individuals to work together to address the root causes of pollution and its adverse effects on allergic individuals and the environment (10). By implementing effective pollution control measures and promoting sustainable practices, we can pave the way for a healthier and more sustainable future for both humans and the planet. Proactive measures today will safeguard the well-being of generations to come, ensuring a harmonious coexistence between humanity and nature (11).

## 2 Outdoor air pollution

Air pollution refers to the presence of harmful substances in the Earth's atmosphere, which can have detrimental effects on human health, the environment, and ecosystems. These pollutants can be either natural or man-made and can take various forms, such as gases, particulate matter, or even biological agents. Outdoor air pollution is commonly recognized to increase the incidence rates of a variety of ailments, including cardiovascular disease, lung cancer, respiratory symptoms, asthma, negatively affected pregnancy, and poor birth outcomes (12). The significant rise in allergies and other prevalent disorders in recent years have been attributed to changes in environmental variables rather than genetic ones (13). Although research on the role of genetic and environmental factors in the development of allergic respiratory diseases is ongoing, there appears to be a link between the rise in the prevalence of allergic airway disorders and an increase in air pollution (14). It is crucial to highlight that an individual's reaction to air pollution is affected by the source and causative agents of pollution in addition with meteorological factors. Some cities are perpetually plagued by black pollution created by automobiles (14). Common outdoor air pollutants include Particulate Matter (PM), Ground-level Ozone (O_3_), Nitrogen Dioxide (NO_2_), Sulfur Dioxide (SO_2_), Volatile Organic Compounds (VOCs) etc. When these pollutants are present in the air, they can interact with pollen grains and other allergens, exacerbating the impact on individuals with allergies. The outdoor air pollution can influence allergies by: increased allergen levels, irritation and inflammation, weakened immune response, new allergy development, and aggravation of existing conditions (14).

### 2.1 Pollution in the open air

The air pollutant is a raw material in the air that can damage the living beings and the climate. The substance can take the shape of solid particles, liquid droplets, or gases, which can be forms aerosol (solid particles or liquid droplets dispersed and carried by a gas). A pollution might be either natural or man-made. Though the nature and concentration of outdoor pollutants varies by location, the most abundant pollutants in metropolitan regions atmosphere are NO_2_, O_3_, and respirable PM. Sulphur dioxide (SO_2_) is another issue in industrial regions. Aeroallergens are conveyed and transmitted by fungal spores or plant-derived particles (pollen, paucimicronic vegetable components, and in certain circumstances soybean dust, for example) (14).

### 2.2 Plant derived allergens or pollen

As a result of global climate change, an increasing number of people are suffering from allergy disorders caused by pollens. As the rate of pollen hypersensitivity in children has recently grown, fast reproducing allergenic plants have emerged as a harmful element for allergic children (15). Aeroallergens, such as pollen grains and fungal spores, cause bronchial constriction in allergic patients, and pollen are commonly employed to investigate the association between air pollution and respiratory atopic illnesses. Pollen grains in the air, paucimicronic plant debris, and pollen grains burst during thunderstorms can all produce allergy symptoms in susceptible individuals (14). Respiratory allergies caused by pollen grain antigens is quite prevalent. Plant-derived respiratory illnesses impact subjects living in cities more than those living in rural settings (14). The identification of pollen allergens in micro aerosol suspensions smaller than pollen grains which could be present in the atmosphere before and after the start of the season, thus lengthening the respiratory symptoms of notified patients, partially explained an etiology of pollen asthma as well as the discordance between pollen count and bronchial symptoms (14). In plants allergens are mostly present in leaves and stems. They could be caused by allergen elution from pollen grains and subsequent dispersion in microdroplets. Furthermore, pollen grain allergens could be transferred to other small particles in the atmosphere, such as DEP, which may get deep into the airways through physical contact or elution (14). Because various pollens are released at different times of the pollen season, allergic persons who are sensitized to more than one type of pollen are in contact for a more extended period of time. Factors impacting pollen distribution also determine the length of the pollen season. A prolonged pollen season and higher levels of air pollen may increase human interaction with allergenic pollens, thus increasing allergy sensitivity (15). Factors like Rainfall, atmospheric temperature, humidity, wind speed, and wind direction can all affect the concentrations of plant pollens and other allergens, determining the incidence of allergic diseases like asthma, allergic rhinitis, allergic conjunctivitis, and even atopic dermatitis. Many studies have shown that increasing levels of carbon dioxide and ambient temperatures increase pollen counts (15). Pollen exposure reduces the immunological response to certain rhinoviruses by lowering the interferon response, regardless of allergy status. According to a global epidemiological investigation, this is also true for SARS-CoV-2 viruses. Mouth-to-nose protection may minimize not just the spread of SARS-CoV-2 viruses, but also pollen intake. Using of mask can be the remedy to this issue, particularly when pollen levels are high (16).

### 2.3 Other outdoor pollutants

Occupational Risks in Seafood Processing: Seafood processing, which takes place on board ships and in land-based factories, shares many risks with farming, such as musculoskeletal strain from heavy labor, exposure to bioaerosols, and seasonal work, which is especially harmful to migrant workers (17). Asthma caused by inhaling shellfish allergens was more clearly reported in a Norwegian fisherman in 1937, and in 6 of 67 workers at a Danish mussel facility in 1944 (18). These and other early studies concentrated on asthma-like skin and airway symptoms (18). Global Impact of Plastic Pollution on Health and Environment: It is becoming more evident that the widespread availability of simple single-use plastic has resulted in a worldwide plastic pollution problem with serious environmental and health effects. Plastic weathering is caused by physical, chemical, and biological processes, that led to the formation of different size of debris ranging in size from micro to nano (19). The situation now is a growing understanding that plastic pieces are spread in the air and can be absorbed by humans, potentially harming the respiratory system and other systems (20).

Soybean Dust and Asthma Outbreaks: Soybean dust was responsible for major asthma outbreaks that were initially attributed to urban air pollution. The findings of research indicating high airborne concentrations on epidemic days and low values on nonepidemic days bolstered the strong link between airborne soybean dust and asthma epidemics (14).

Ubisch Bodies—A Minor Allergen Carrier in Plants: Ubisch bodies are another minor allergen carrier. Many higher plants have anthers with these spheroidal structures that develop with pollen exine. Ubisch bodies may be involved in pollen distribution, and their size is ideal for penetration into lower airways (14).

Asian Sand Dust and Allergic Respiratory Complications: Although Asian sand dust particles moved from the Asian continent as micrometer-sized particles cause respiratory system injury, we already knew that when the particles coexist with an allergen, allergies such as bronchial asthma and allergic rhinitis worsen (13).

Impact of Air Pollution on DNA Methylation in NOS Genes and Respiratory Health in Children: Observations reveal that the cooperation between particulate matter (PM2.5 and PM10) air pollution and DNA methylation in NOS genes, which play a part in nitric oxide (NO) homeostasis. The exploration suggests that air pollution affects DNA methylation in NOS genes, impacting NO regulation and potentially impacting respiratory health issues in children, particularly those with asthma and wheeze. It is set up that PM2.5 exposure was linked to reduced NOS2A promoter methylation, potentially increasing inducible NO synthase iNOS) expression, while PM2.5 exposure influences the extensive methylation of DNA in the promoter of NOS3, leading to reduced transcriptional exertion and lower NO product (21). The aftereffects indicate that DNA methylation fluctuations in NOS genes may contribute to asthma pathogenesis and respiratory inflammation. still, the counteraccusations of these changes for health issues are complex and require farther examination in applicable tissues and longitudinal studies (21). Different types of air pollutants are linked to allergic conditions and several illnesses as outlined in Table 1.

**Table 1:** List of various air pollutants are associated with allergic conditions and diseases.

Air pollutants	Sources	Associated diseases	References
(i) Ammonia (NH3)	Agricultural activities (fertilizers) Livestock manure	Respiratory irritation	(22)
(ii) Carbon Monoxide (CO)	Industrial processes Incomplete combustion of fossil fuels	Headaches, Dizziness,	(23)
(iii) Nitrogen Dioxide (NO_2_)	Vehicles, stoves, furnaces Combustion of fossil fuels (power plants, industrial processes)	Reduced oxygen delivery to tissues Respiratory irritation Exacerbation of asthma Increased susceptibility to respiratory	(14)
(iv) Nitric Oxide (NO)	Combustion of fossil fuels (vehicles,	Respiratory irritation, Infections	(14)
(v) Sulphur Dioxide (SO_2_)	power plants. industrial processes Combustion of fossil fuels (vehicles, power plants, industrial processes)	Respiratory irritation Exacerbation of asthma.	(24)
(vi) Volatile Organic Compounds (VOCs)	Motor vehicle exhaust Industrial emissions Solvent uses.	Aggravation of cardiovascular diseases Eye, nose, and throat irritation Headaches & dizziness	(25)
(vii) Ozone (O_3_)	Consumer products (paints, etc.) Formed by chemical reactions involving VOCs and NOx in the presence.	Respiratory irritation, Aggravation of asthma.	(14)
(viii) Particulate Matter	Sunlight Combustion processes (vehicles, industrial activities, construction, agricultural activities)	Reduced lung function Aggravation of respiratory conditions Cardiovascular disease	(26)

## 3 Indoor air-pollution

Indoor air pollution describes to as the presence of harmful pollutants and contaminants in our surroundings air inside buildings or enclosed spaces. Harmful pollutants can derive from various sources, including building materials, household products, combustion processes, and outdoor pollutants that enter the building. Indoor air pollution can have downside effects on human health, leading to respiratory problems, allergies, and other illnesses also causing deaths.

### 3.1 Common origins of indoor air pollution which we don't care in daily life

Particulate matter (PM): Pm is referred as carbonaceous particles along with organic chemicals and reactive metals which come from outdoor environment and some indoor activities like cooking purpose, fossil fuel combustion activities, machine operation (laptop, air conditioner) and residential hobbies (4).

VOCs: Volatile organic compounds (VOCs) are gases containing a variety of components or chemicals released from liquids or solids. VOCs are casted out from various sources like wall stains, pesticides, paints, varnishes, solvents, waxes, adhesives, wood preservatives, cleansers, fuels, plastics, copy machines, printers, perfumes, air fresheners, building materials and furnishings (4).

NOx: NOx mainly includes two types of gases nitric oxide (NO) & nitrogen dioxide (NO) eject from cooking heaters & stoves. OZONE: O_3_ is a dangerous hazardous agent produced from O_2_ & NOx by photochemical reactions in the atmosphere. The common machines in our home emitting indoor O_3_ gas include photocopiers, disinfecting devices, computers, air-purifying devices, and other official uses devices (27).

SOx: SO_2_ leads to respiratory tract infection, eye and skin irritation. Indoor SO_2_ comes from vented gas appliances, oil furnaces, kerosene heaters, coal or wood stoves, tobacco smoking. TOXIC METALS: Due to some open building construction heavy metals such as Co, Al, Cu, Ni, As, Pb, Cr, Fe, Zn etc. which not only effects on inhalation also cause dermal allergic effects. AEROSOLS: Like corona we know this transmits by biological aerosols also few bacteria, fungi, spore, virus spread through this (28).

Ventilation and air conditioner: Due to lack of horizontal ventilation indoor air pollutants don't go away from a room which increase poor air quality also due to air conditioner gives more humidity which leads grow of fungi and mites which causing allergic reactions. Radon: Radon is a naturally occurring radioactive gas which can seep into buildings through cracks in the foundation, walls, or floors. Daily exposure of radon can increase the risk of lung cancer (4).

Pesticide: Pesticides are used to control for pests, including fungi, bacteria, insects, rodents, and other organisms which are semi-volatile to impact on skin and eye irritation, dizziness, headaches, nausea, cancer, asthma, diabetics.

## 4 Food allergy

Food allergy is an inappropriate immunological response to food. A food allergy sufferer's immune system recognizes the allergenic food's proteins as dangerous invaders and sets off an allergic reaction when they consume it or come into touch with it. The immune system releases histamine in response to the allergenic proteins, which causes a variety of allergy symptoms (29). The allergic reactions symptoms might be moderate to severe. After exposure, this often happens minutes to hours later. They could include hives, vomiting, diarrhea, vomiting, swollen tongue, itching, difficulty breathing, and low blood pressure. The condition is referred to as anaphylaxis (it's Life-threatening symptoms or signs can be happened by Constrict and tighten the airways) when the symptoms are severe (30). Proteins found in common daily foods such as milk, eggs, wheat, fish, and others are responsible for causing allergic reactions in some individuals. These routine foods have the potential to trigger allergies due to the presence of specific proteins. Allergen found in common daily foods are listed in Table 2.

**Table 2:** Allergen found in common daily foods.

Foods	Allergen present	References
(i) Milk	Casein, Lactose	(31)
(ii) Eggs	Ovalbumin, Ovomucoid	(32)
(iii) Peanuts	Ara h1, Ara h2	(33)
(iv) Tree nuts	Almonds: Pru du 6 and Pru du 8. Walnuts: Jug r 1 and Jug r 3 Cashews: Ana o 1 and Ana o 2 Pecans: Car i 1 Hazelnuts (Filberts): Cor a 1 Brazil nuts: Ber e 1 Pistachios: Pis v 1 and Pis v 3 Macadamia nuts: Mac i 1	(34)
(v) Wheat	Gliadins, Glutenins	(35)
(vi) Soy	Gly m 5, Gly m 6	(32)
(vii) Fish	Parvalbumin, Tropomyosin, Arginine kinase	(32)
(viii) Eggplant	Profilin, Bet v 1 homologues	(36)

### 4.1 Food allergies vs. food intolerance

It's important to know the difference between food allergies and food intolerance. Food allergy is different from food intolerance on the basis that is produced by viruses or toxins found in food, additionally from so-called food intolerance, which exhibits comparable symptoms but different pathogenic mechanisms. When the body struggles to disintegrating or processing dietary components, it can cause food intolerance, a non-allergic unpleasant reaction to those foods (37). Food intolerances are not life-threatening and do not engage the immune system like food allergies do. Instead, they are frequently brought on by enzyme deficits or susceptibility to specific dietary components (38). Food allergies represent a significant public health risk that has a substantial impact on the lives of allergic patients and their families. It is becoming more common in urbanized areas. Because of its rising frequencies and adding health care application, food allergy (FA) has turn a estate of growing interest for patients, physicians, and policymakers around the public. Food allergy (FA) has been reported to affect people of all socioeconomic and demographic backgrounds, involving patients of all eras (39). Both with respect to primary immunoglobulin E(IgE)- intermediated food hypersensitivity and other food- activated provisions that run through a heterogeneity of immunologic mechanisms (e.g., pollen- FA syndrome, food protein- convinced enterocolitis development, eosinophilic esophagitis (EoE) (40). The need to more infer the disbursement of FA among grown-up populations is increasingly critical because potent allergy precluding and treatment modalities are arising, which overwhelmingly target pediatric populations. FA is thought to have an impact around 220 million individuals worldwide (41).

### 4.2 Food allergens

Food allergens are chemicals found in food that, in some people, might cause an allergic reaction. Food allergies can appear in several ways, such as additives, components, or cross-contamination during manufacturing or preparation (42). Because food manufacturers are frequently compelled to prominently mark allergens on their products, the individuals who possesses food allergy can more easily recognize potential dangers (43). As “big eight” the most typical food allergies are frequently listed as follows:

(i) Milk: It is beneficial to our health and to the strength of our bones. Additionally, it's one of the most typical dietary allergies, particularly in kids. Surprisingly it's observed that 2% to 3% of kids under the age of three have milk allergies. Recent findings disprove the assumption that most children will outgrow this allergy by the time they were 3 that was once widely held by professionals (31). Less than 20% of youngsters in one research had outgrown their allergy by the age of four. However, before the age of 16, over 80% of youngsters will likely outgrow their milk allergy (31).
(ii) Eggs: One might be sensitive to eggs if he/she experiences hives or other physical responses after eating eggs. Egg allergies are among the most prevalent allergies, particularly in children. When the body's defense system excessively interacts to the proteins in egg whites or yolks, egg allergy results (32). When we consume eggs, our body perceives the egg protein as an external intruder, and releases chemicals to fight it. These substances result in allergic response symptoms (32).
(iii) Peanuts: Among the most prevalent food allergies associated with anaphylaxis, peanuts or groundnuts are mostly responsible edible seeds which causes potentially lethal disease that demands quick attention and treatment (33). Peanut allergy awareness in youngsters has grown in recent years, as has the number of documented instances. Peanuts are legumes (the same family as soybeans, peas, and lentils), not tree nuts. While it was previously thought that a peanut allergy was permanent, research has shown that up to 20% of people with a peanut allergy eventually outgrow it (33).
(iv) Tree nuts: Tree nuts, along with peanuts and shellfish, are among the food allergens most frequently linked to anaphylaxis, a dangerous, rapid-onset allergic reaction that can be fatal. A tree nut allergy usually lasts a lifetime; only around 10% of people with this allergy grow out of it. Peanuts and tree nuts are frequently confused (34). Although peanuts are legumes rather than nuts and studies show that 25% to 40% of people who are allergic to peanuts also have an allergy to at least one tree nut. Seeing an allergist is the best approach to clear up any confusion and manage your tree nut allergy (34).
(v) Wheat: If one acquires hives or a rash after eating cereal, bread, or pasta, or if one experience a stomachache, or if one's nose feels stuffy or runs, then he or she may have a wheat allergy (35). Wheat allergies, like high fever and other allergies, emerge when the immune system overreacts and becomes sensitized to wheat, but it is normally harmless to most people (35).
(vi) Soya: Soya legume, is a major ingredient in infant formulae and many other processed foods. There are many dietary allergies in young children, among them “soy” is one of the most prevalent. Allergies typically emerge in newborns and in young kids under the age of three, and many outgrow the allergy during childhood (32).
(vii) Fish: An allergy to finned fish, while less common in the general population than other types of food allergies, is a common cause of anaphylaxis, a potentially life-threatening allergic reaction that arises suddenly, affects breathing, and can put the body into shock (44). There are many food allergies which are often discovered in infants and children under 3 years age, but in case of the allergies which arise due to fish consumption might not be discovered until maturity stage; in one study, up to 40% of those who narrated a fish allergy faces no trouble of fish consumption until they reached adulthood. If one gets hives or a stomachache after eating crab, lobster, or other shellfish, he or she may have a shellfish allergy. Shellfish also reported as a prominent allergen constituent for dietary allergies. A shellfish allergy is distinct from a fish allergy. Those who are allergic to shellfish should not avoid fish, and vice versa (44).
(viii) Eggplant: Allergies to eggplant, also known as aubergine, are uncommon. Eggplants are a member of the nightshade plant family, which includes plants that contain alkaloids (36). People who are allergic to eggplant should be aware that the presence of eggplant in a meal may not be visible. Eggplant allergies are most frequent in youth, but they can develop at any age (36).

### 4.3 Some other allergy causative foods

Other than the above-mentioned food there are several other allergy causing foods like celery, asparagus, avocado, bell pepper, cabbage, carrot, fennel, lettuce, potato, pumpkin, turnip, and zucchini (42). An investigation of the fruits that cause allergies yields a list of 12–15 fruits that are commonly related which include apple, peach, kiwi, musk melon, grape, cherry, strawberry, banana, mango, and pomegranate. The majority of these are widely available in vegetable and fruit markets worldwide; nevertheless, a few unusual foods, particularly tropical fruits, and berries, have been shown to trigger allergies in susceptible individuals (45). Pollen-food disorders are interconnected with various plants. There are many fruits borne syndromes, among them Birch-fruit-vegetable syndrome is widely observed. Foods from the Rosaceae family, such as apple, pear, peach, and almond, are the most prevalent triggers for birch-allergic patients. Another is celery-birch-mugwort-spice syndrome, which occurs when celery pollen reacts with both birch and mugwort pollens (45).

### 4.4 Allergy and genetically modified (GM) food

The allergenicity and toxicity of GM foods are the two main issues about their safety, where allergenicity could have developed in a variety of ways (46, 47). Genetic engineering may have resulted in the creation of a novel protein, the introduction of a known allergy, or the enhancement of a GM crop's innate tendency to induce allergies (48). Two publicly documented incidences of allergenicity in GM food studies fueled speculation that they may be contributing to the worldwide surge in allergies. The first, in 1966, involved the incorporation of a Brazil-nut protein into a soybean to increase the nutritional value of the soya bean (48). In addition, an allergenic protein was delivered, causing an allergic reaction in human subjects. This product was never approved for sale. The second, in 2005, involved mouse studies in which a bean designed to resist pea weevil caused an immunological response in the animals' lungs (49). These examples are frequently used to bolster concerns that GM technology is risky and unpredictable. An alternate view is that safety testing was effective in both circumstances prior to product release (49).

## 5 Health issues associated with pollution

**Figure 2:**
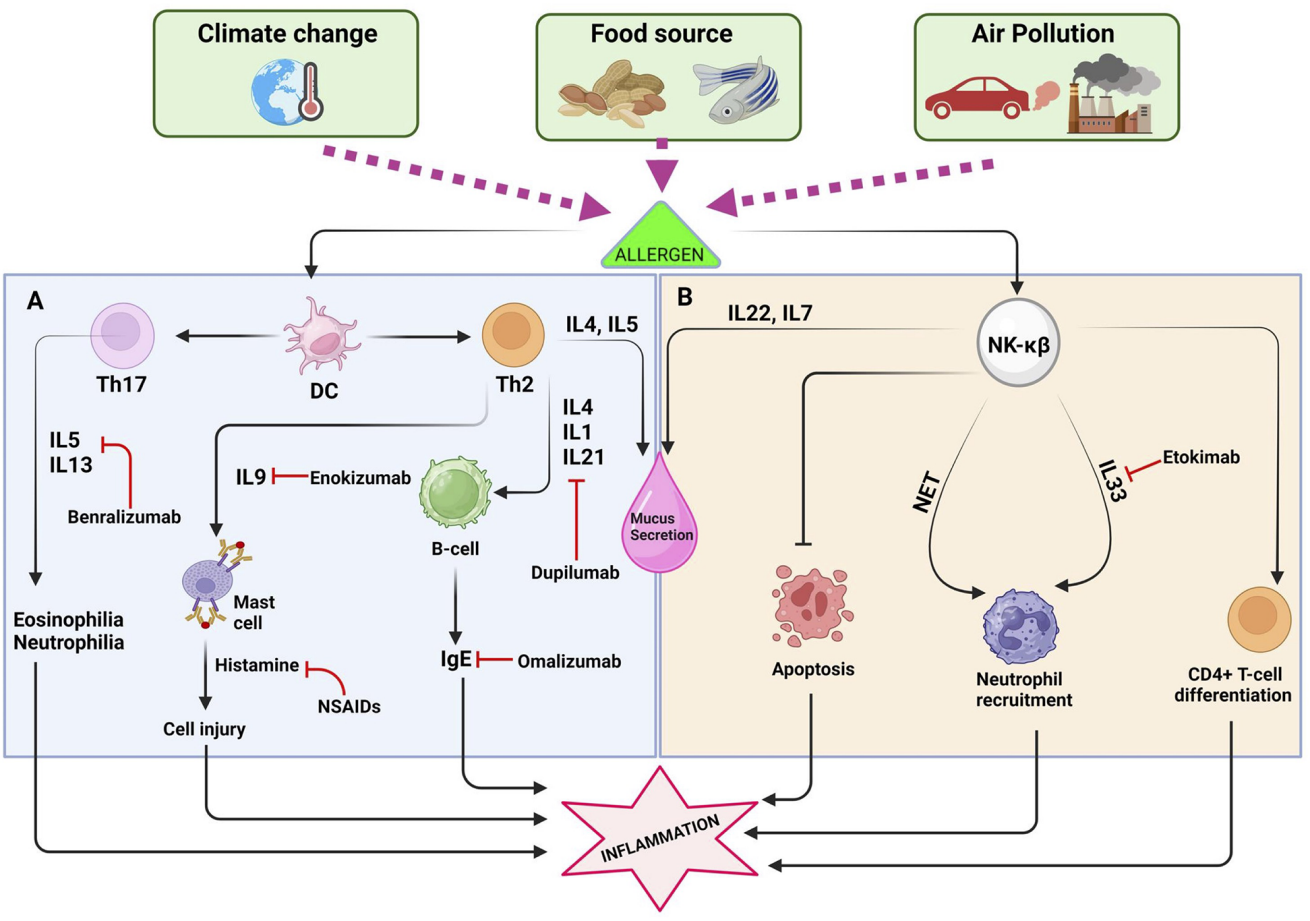
The interaction between dendritic cells (DCs) and the T-helper cell, along with the NK-KB pathway, plays a crucial role in promoting inflammatory reactions. **(A)** Exposure to allergens triggers an intricate interaction between dendritic cells (DCs) and T-helper cells, culminating in the production of a spectrum of cytokines including IL-13, IL-4, IL-5, IL-33, IL-9, and IgE. These cytokines play pivotal roles in orchestrating immune responses, leading to detrimental effects such as cell damage, excessive mucus production, and the initiation of severe inflammatory processes. Consequently, pharmaceutical interventions target these cytokines to alleviate symptoms and mitigate allergic reactions effectively. **(B)** The NF-κB pathway plays a significant role in the development and progression of allergies by orchestrating complex immune responses. One of its key functions is the recruitment of neutrophils, which are crucial for combating pathogens but can also contribute to tissue damage during allergic reactions. Additionally, NF-κB activation leads to the stimulation and activation of various other immune cells, amplifying the inflammatory cascade characteristic of allergic responses.

Air pollution is a major environmental and public health concern, as it can have significant serious effects on human health. It is caused by the release of harmful substances into the air, primarily from human activities such as burning fossil fuels, industrial, processes, transportation, and some indoor air pollutants. The schematics presented in Figure 2, highlighting the severe health effects of pollution and strategies for prevention through therapies, are of utmost importance.

### 5.1 Allergies and immune system

In medical terms, an allergy is a hypersensitivity reaction of the immune system to a normally harmless substance known as an allergen. When a person with an allergy encounters an allergen, their immune system reacts excessively, leading to various symptoms such as sneezing, itching, swelling, rash, or respiratory issues. Allergies can be triggered by a wide range of substances, including pollen, pet dander, certain foods, medications, oral care products and insect venom (50–53).

Managing allergies often involves avoiding triggers and may include medications or allergy shots (immunotherapy) under the guidance of a healthcare professional (54). Allergies are known to be the consequences of the immune system's response to allergens, that are components perceived by the body as harmful even though they may be harmless to most people. The immune system protects our body against the invasion of detrimental pathogens like bacteria and viruses. In the case of allergies, body's defense mistakes allergens, such as pollen or certain foods, as threats and launches an immune response (55). When a person with allergies encounters an allergen, the immune system produces antibodies called immunoglobulin E (IgE). These antibodies trigger the release of histamine and other chemicals, leading to allergy symptoms like sneezing, itching, swelling, and inflammation (56). In some cases, the immune system's response to allergens can be severe and lead to life-threatening allergic reactions, such as anaphylaxis. Managing allergies often involves identifying and avoiding triggers, taking medications to alleviate symptoms, and, in certain cases, undergoing immunotherapy to desensitize the immune system to specific allergens. Consulting with a healthcare professional is crucial for proper diagnosis and management of allergies. Certain allergic diseases that were initially predominated to Western civilization lifestyle have now started to invade Asian people's life too. The probable reason could be the global influence of westernization of communal cultures and the migration to western countries. Atopic diseases have environmental influences (57). If migrating to western countries is considered it has been noticed that individuals who are developing atopic diseases show a degree of varying difference of extent of the diseases based on the time of residing to those particular places or the time of migration (58). Along with migration, other things are related to disposition to allergic conditions like for individuals belonging to different families and how big or small they are, food habit, lifestyle differences also come in consideration (59).

### 5.2 Allergic rhinitis

The contribution of childhood DNA methylation in the coalition of risk elements of juvenile period of life and allergic rhinitis is studied (60). In this study a patient group of age 6 years is recruited and divided into two subgroups, one of patients having allergic rhinitis and another of patients without allergic rhinitis. The DNA methylation patterns of the IFN-γ promoter regions in CD4^+^ cells were analyzed using bisulfite sequencing. The percentage of Th1 was investigated by flow cytometry. The relationship among DNA methylation, early-life environment, and AR (Allergic Rhinitis) was examined (60). After using different statistical parameters like maternal health conditions of the children (mothers having allergies or not), birth seasons and interior S of the rooms they live in it was concluded that the early life environment could possibly act as a likely risk component conducive to allergic rhinitis. IFN-γ Y methylation plays the role as a mediator of the effect of birth season on allergic rhinitis (61).

### 5.3 Urbanization and its impact on the condition of allergic rhinitis and bronchial asthma

In both the cases allergic rhinitis and bronchial asthma are fruited because of residential impact. Urbanized areas are more prone to be exposed to such allergic conditions than rural areas (62). In years millions of people are affected by asthma worldwide. Reported cases of asthma is prevalently higher in developed countries (63). The cytokines play major roles in asthmatic conditions. In this regard, Th2 cytokines (IL4, IL2) are proven to be the major contributors of asthmatic conditions whereas interferon-gamma, the Th1 cytokine mediates the early inflammatory response of chronic asthma (64). Health hazards associated with allergy like allergic rhinitis and asthma are associated with urban/rural living and people living in urban areas have a greater tendency to be exposed to allergic rhinitis and asthmatic conditions (65).

### 5.4 Deterioration of asthmatic conditions due to environmental pollution

Diesel exhaust particles, a major component of fine particulate matter (PM2.5) are the contributors to upsurge of allergic diseases such as asthma (66). Moreover, when the allergens are present, DEPs increase the local (lung) expression of cytokines and bronchial asthma is highly triggered by the enhanced expression of interleukin (IL)-5, which is produced by Th2 lymphocytes activates eosinophils. Asthma being an airway inflammatory disease is prevalently dominated by air pollution in its every possible form. Fine particulates are the being the matter of huge concern regarding air pollution and as the consequence a worsen condition of allergic asthma (67).

### 5.5 Cardiovascular diseases

Fine particulate (PM2.5) air pollutant responsible for CVD. They may lead damage or dysfunctional endothelium & result to atherogenesis, thrombosis and the incidence of myocardial infarction, stroke, and sudden cardiac death (68). Traffic-related air pollution (TRAP) contains multiple air pollutants (PM2.5) become more modify & formation of coronary artery calcium (69). In a study, post-menopausal US Women who are daily contacted with NOx. They are correlated with higher risk of hemorrhagic stroke & ischemic stroke (70).

### 5.6 Respiratory diseases

Respiratory diseases, including emphysema (a component of COPD) and chronic bronchitis, rank as the fourth leading cause of death in the United States and the third leading cause globally. These conditions are exacerbated by the presence of pollutants such as ozone (O_3_), nitrogen oxides (NOx), sulfur oxides (SOx), and fine particulate matter (PM2.5) in the air. These harmful pollutants contribute to the development and worsening of respiratory ailments, leading to increased morbidity and mortality rates. Efforts to reduce emissions of these pollutants are essential in mitigating the burden of respiratory diseases and improving public health worldwide (71, 72).

### 5.7 Neurological disorders

Neurological disorders such as Parkinson's disease and Alzheimer's disease prone to progress more rapidly in individuals exposed to poor-quality air. Living in environments with elevated levels of pollution exacerbates cognitive decline and memory loss associated with these conditions. Studies have shown a disturbing correlation between air pollution and the advancement of neurological diseases (73). Particularly concerning are the effects on memory retention and cognitive function. Individuals residing in areas with heightened pollution levels not only experience more pronounced symptoms but also exhibit a hastened progression of these debilitating conditions. Therefore, addressing pollution not only has environmental benefits but also plays a crucial role in mitigating the burden of neurological disorders. Efforts to reduce air pollution are essential not only for promoting overall public health but also for safeguarding cognitive function and memory in vulnerable populations.

### 5.8 Cancer

Due to poor quality air associates with various oxidative stress elements which leads to inflammation in human cell and causes for chronic disease and cancer. Long term high exposure of NOx & NO_2_ risks for breast cancer (74). Presence of methylene chloride, polycyclic organic matter, propylene dichloride, and styrene in surrounding air are associated with ER Positive breast cancer (75). Benzene is highly exposure from petroleum industry, rubber manufacturing, automobile repairing, gasoline, cigarette. Benzene widely responsible for leukemia with Non-Hodgkin Lymphoma and chromosomal changes disorder (76). In Coal-Fired power plants workers and surrounding people become a victim for lung cancer (77).

## 6 Generalized treatments of allergies

### 6.1 Allergen immunotherapy

Allergen immunotherapy, also termed as allergy shots or desensitization therapy, is a time-tested and effective treatment for allergic diseases. A comprehensive review of allergen immunotherapy is presented by exploring its mechanisms of action, clinical applications, safety profile, and prospects. As a disease-modifying therapy, allergen immunotherapy offers long-term relief from allergic symptoms and has the capacity to alter the inherent progression of allergic diseases (78). Allergic diseases, such as allergic rhinitis, asthma, and atopic dermatitis, are prevalent and pose a significant burden on global health. Allergen immunotherapy stands as a promising treatment modality, offering a targeted approach to address the underlying cause of allergies (78). Allergen immunotherapy involves the administration of gradually increasing doses of allergens to allergic individuals. This exposure aims to induce immune tolerance, shifting the immune response from an allergic to a non-allergic state. As a result, the body becomes desensitized to the allergens, leading to reduced allergic symptoms upon subsequent exposures (79). Allergen immunotherapy is effective in managing various allergic conditions, involving allergic rhinitis, allergic asthma, stinging insect allergies, and atopic dermatitis. It is notably advantageous for who do not achieve satisfactory relief with pharmacological treatments or encounter adverse reactions to medications (80). Two primary forms of allergen immunotherapy are subcutaneous immunotherapy (SCIT) and sublingual immunotherapy (SLIT) (80). SCIT involves injections under the skin, while SLIT uses allergen extracts administered as drops or tablets under the tongue. Both forms have demonstrated efficacy and are suitable different patient populations (80). Unlike symptomatic treatments, allergen immunotherapy acts as a disease-modifying therapy, altering the natural course of allergic diseases (81). Long-term benefits may persist even after the discontinuation of treatment, making it a valuable investment in patients' health (81). Allergen immunotherapy is generally safe when administered by trained healthcare professionals (82). However, it carries a risk of allergic reactions, which can range from mild local reactions to rare systemic reactions. Proper patient selection, individualized treatment plans, and close medical supervision are essential to mitigate these risks (82).

Ongoing research aims to optimize allergen immunotherapy by exploring novel formulations, adjuvants, and delivery methods (83). The emergence of biotechnological advancements, such as allergen epitope mapping and engineered allergens, holds promise for further enhancing treatment efficacy and safety (84). Successful allergen immunotherapy requires a collaborative approach between healthcare professionals and patients. Comprehensive patient education, realistic expectations, and shared decision-making empower patients to actively participate in their treatment journey (85). Allergic conditions, ranging from seasonal allergies to food intolerances, affect millions of individuals worldwide, impacting their quality of life and overall health. Traditional treatments like antihistamines and immunotherapy have proven effective but often require multiple sessions and extended periods for significant relief. The emergence of intra-lymphatic immunotherapy (ILIT) offers a promising breakthrough in allergic treatment (86). Intra-lymphatic immunotherapy is a cutting-edge procedure designed to modify the immune system's response to specific allergens. Unlike conventional subcutaneous or sublingual immunotherapy, where allergens are administered through injections or under the tongue, ILIT involves direct injection of allergens into the lymph nodes (86). During an ILIT session, a healthcare professional injects a precise amount of allergen extract into the patient's lymph nodes. This triggers a controlled immune response, resulting in the generation of regulatory T-cells and other immune cells that reduce the body's allergic reactivity to the specific allergen (87). One of the key advantages of ILIT lies in its efficiency. Compared to traditional immunotherapy, which involves multiple injections over several years, ILIT typically requires only a few sessions, usually spaced several weeks apart. This accelerated treatment approach can lead to faster symptom improvement and greater patient compliance (86). ILIT allows for personalized treatment plans tailored to each patient's specific allergens. Healthcare professionals can target the most relevant allergens for everyone, optimizing treatment efficacy and tailoring the therapy to their unique needs (87).

ILIT offers several potential benefits over traditional treatments, including quicker onset of symptom relief, reduced treatment duration, and fewer clinic visits. Moreover, it may be a viable option for individuals who have not experienced satisfactory results with other allergy treatments (87). While ILIT has shown promising results, it is essential to consider individual patient factors and potential risks. Like any medical procedure, ILIT requires proper evaluation and supervision by qualified healthcare professionals to ensure safety and efficacy (88).

As ILIT gains attention in the medical community, ongoing research aims to further understand its mechanisms and optimize treatment protocols. Future developments might include the expansion of allergens that can be targeted through ILIT and its application to various allergic conditions (88).

**Figure 3:**
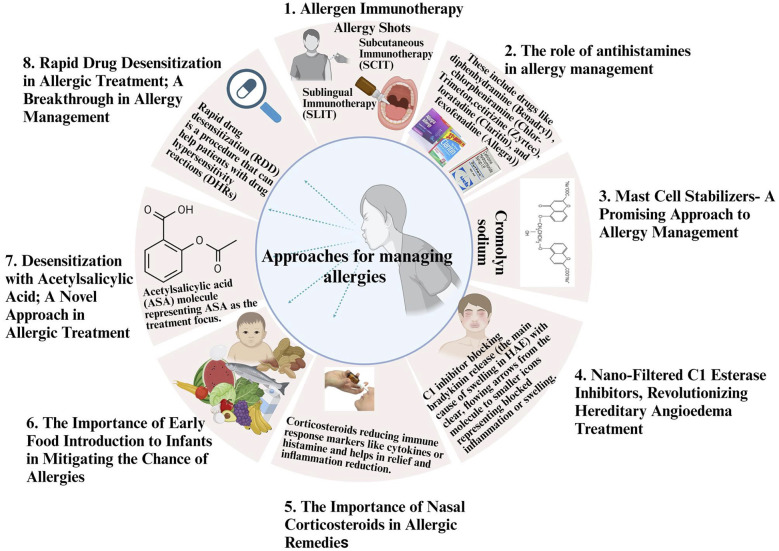
Approaches for managing allergies: overview of strategies in allergy management, including (1) allergen immunotherapy, (2) antihistamines, (3) mast cell stabilizers, (4) nano-filtered C1 esterase inhibitors for hereditary angioedema, (5) nasal corticosteroids, (6) early food introduction for infants, (7) desensitization with acetylsalicylic acid, and (8) rapid drug desensitization.

Allergic conditions, particularly food allergies, have become a growing concern, affecting millions worldwide and significantly impact the standard of living for affected individuals. In recent years, low-dose oral immunotherapy (LDOIT) has established as an innovative treatment option, offering the ability to desensitize the immune system to allergens gradually**.** While LDOIT shows promise as a therapeutic strategy, careful consideration of individual patient factors and expert medical supervision are essential for safe and successful implementation (89). LDOIT involves administering small, gradually increasing doses of the allergen orally to induce immune tolerance. This process aims to retrain the immune system to recognize the allergen as harmless, thus reducing allergic reactions upon subsequent exposures (89). Clinical studies unveiled favorable outcomes in desensitizing individuals with food allergies through LDOIT. Patients have experienced reduced allergic reactions to allergenic foods, improved quality of life, and increased tolerance to previously reactive substances. While LDOIT holds significant potential, it is not without risks (90). Adverse reactions, including allergic responses during the dose escalation phase, can occur and necessitate close medical supervision. LDOIT should only be conducted under the guidance of qualified allergists or immunologists (91). Not all individuals with allergies may be suitable candidates for LDOIT. Factors such as the type and severity of the allergy, age, medical history, and patient preferences must be considered to determine eligibility for this therapy (91). The long-term effects of LDOIT remain an active area of research. Questions persist regarding the duration of protection, possible demand for continuous care, and whether immune tolerance can be sustained after the therapy is discontinued (91). LDOIT shows promise beyond food allergies. Researchers are investigating its' potential for treating other allergic conditions, such as allergic rhinitis, asthma, and insect venom allergies. These efforts expand the horizons of LDOIT as a versatile therapy in allergy management (89). Despite its potential, LDOIT faces challenges concerning standardization, widespread availability, and addressing patient anxiety about adverse reactions. Further research and collaboration between healthcare professionals and regulatory bodies are vital to overcome these obstacles (89) (Figure 3).

### 6.2 The role of antihistamines in allergy management

Antihistamines, known for their effectiveness in treating allergic rhinitis, common cold, and influenza, have become a popular choice for individuals seeking relief from seasonal allergies (92). These over the counter, generic drugs provide a cost-effective solution to alleviate symptoms like nasal congestion, sneezing, and hives triggered by pollen, dust mites, or animal allergies. While antihistamines offer short-term relief with minimal side effects, it is critical to comprehend their limitations and potential health risks (92).

Antihistamines work by blocking histamine, a chemical released during an allergic reaction, thereby reducing the unpleasant symptoms caused by allergens (93). Their wide availability and affordability make them a convenient choice for managing acute allergy episodes, providing rapid relief from discomfort (93). It is generally considered safe under the guidance of a healthcare professional. However, it's crucial to be aware of potential side effects such as drowsiness, dry mouth, and blurred vision. Chronic allergies left untreated can lead to more severe health problems, such as asthma, sinusitis, and lower respiratory tract infections. While antihistamines can alleviate immediate symptoms, they may not address the root cause, leaving individuals at risk of developing long-term complications (94) (Figure 3).

### 6.3 Mast cell stabilizers- a promising approach to allergy management

Mast cell stabilizers, a class of medications designed to prevent the efflux of histamine and other inflammatory mediators, have emerged as effective tools in managing allergic reactions (94). By stabilizing the mast cell membrane, these agents provide relief from allergy symptoms and are particularly beneficial when used before exposure to allergens (95). Among the available options, sodium cromoglycate (cromolyn sodium) stands out as a widely accessible intranasal over-the-counter drug, offering a first-line treatment for mild allergic reactions and a valuable complement to other regimens (96). Sodium Cromoglycate; A Versatile Over-the-Counter Option: Sodium cromoglycate, known for its mast cell-stabilizing effects, has been available in an intranasal format without a prescription for nearly a decade (97). This drug is a first-line choice for managing mild allergic reactions, providing relief from symptoms triggered by allergens. Additionally, it serves as an effective add-on treatment when combined with other regimens to enhance overall allergy management (98). Nedocromil: Nedocromil, a potent mast cell stabilizer, is currently only FDA-approved in the USA as an inhaled preparation for asthma and a topical ophthalmic solution. Despite its limitations in terms of available forms, nedocromil showcases the potential for future expansion into other treatment avenues (99).

#### 6.3.1 Ophthalmic mast cell stabilizers

Apart from cromolyn sodium and ketotifen, other ophthalmic mast cell stabilizers like olopatadine demonstrate their effectiveness in managing ocular allergy symptoms. Furthermore, topical antihistamines, such as olopatadine, have been found to possess mast cell-stabilizing properties, offering additional options for managing allergies that affect the eyes (100). The Promise of lodoxamide and Future Prospects: lodoxamide, another mast cell stabilizer, shows promising results in ongoing studies, potentially paving the way for FDA approval in the future. As the landscape of allergy management evolves, novel drugs like lodoxamide hold the promise of broadening treatment options for individuals suffering from allergies (101).

### 6.4 Nano-filtered C1 esterase inhibitors, revolutionizing hereditary angioedema treatment

Hereditary angioedema (HAE) is a rare and potentially life-threatening genetic disorder characterized by recurrent episodes of localized swelling, commonly affecting the face, limbs, and gastrointestinal tract. Traditionally, C1 esterase inhibitors have been a cornerstone of HAE treatment, effectively preventing uncontrolled activation of the complement system. The advent of nanotechnology has now paved the way for a groundbreaking development: nano filtered C1 esterase inhibitors (102). HAE results from a deficiency or dysfunction of the C1 esterase inhibitor protein, leading to an excess of bradykinin, a potent vasodilator. This imbalance triggers swelling in various body parts and can be life-threatening when it affects the airway. Traditional C1 esterase inhibitors, administered intravenously, aim to restore the balance of C1 esterase inhibitor protein, mitigating the severity and frequency of HAE attacks (103). Nano-filtered C1 esterase inhibitors mark a significant advancement in HAE treatment. By leveraging nanotechnology, these therapies achieve highly targeted delivery, enhanced bioavailability, and prolonged circulation time in the bloodstream. These attributes optimize the effectiveness of C1 esterase inhibition, potentially reducing the required dosage and frequency of administration (104). Nano-filtered C1 esterase inhibitors boast improved drug stability, reducing the risk of protein degradation and ensuring consistent therapeutic efficacy. Moreover, their targeted delivery minimizes off-target effects, enhancing the safety parameters of these therapies and potentially reducing the threat of adverse reactions (104). The application of nanotechnology in HAE treatment exemplifies the power of precision medicine. Nano-filtered C1 esterase inhibitors can be tailored to individual patients, optimizing dosing and treatment regimens based on specific genetic and clinical factors. This customized method holds immense promise for optimizing outcomes and elevating the well-being of HAE patients (102).

The accessibility of nano-filtered C1 esterase inhibitors may revolutionize HAE treatment accessibility. With the potential for subcutaneous administration, these therapies could allow patients greater independence and convenience in managing their condition, leading to improved treatment adherence and overall health outcomes (105, 106). As nano-filtered C1 esterase inhibitors continue to undergo rigorous clinical evaluation and refinement, the landscape of HAE treatment is set to transform significantly. Ongoing research seeks to explore novel drug delivery methods, combination therapies, and innovative applications of nanotechnology to unlock the full potential of these advanced treatments (107).

### 6.5 The importance of nasal corticosteroids in allergic remedies

Allergic rhinitis, commonly known as hay fever, affects a considerable segment of the global population, causing discomfort, sneezing, nasal congestion, and itchy eyes. Though there are multiple treatments available for allergic symptoms, nasal corticosteroids have evolved as a cornerstone in allergy management (108). Allergic rhinitis is an allergic reaction to airborne allergens, such as pollen, pet dander, or dust mites. Nasal corticosteroids, a class of medications, are specifically designed to reduce inflammation and alleviate symptoms associated with allergic rhinitis (108). Nasal corticosteroids work by inhibiting the release of inflammatory substances and reducing the swelling of the nasal passages. These medications target the immune response responsible for allergic reactions, effectively curbing symptoms like sneezing, nasal itching, and congestion (109). Nasal corticosteroids are highly effective in managing both seasonal and perennial allergic rhinitis. They provide superior symptom relief compared to antihistamines alone, making them a preferred choice for individuals with moderate to severe allergies (110). One of the significant advantages of nasal corticosteroids is their suitability for long-term use. Unlike some allergy medications, nasal corticosteroids might be utilized continuously, maintaining symptom control, and boosting the living conditions for allergy sufferers (110). Since nasal corticosteroids are applied directly to the nasal passages, they have minimal systemic absorption, resulting in a reduced risk of common side effects associated with oral corticosteroids, such as weight gain, mood changes, or osteoporosis (110). For moderate to severe allergic rhinitis, medical guidelines often recommend nasal corticosteroids as a first-line treatment. They are considered particularly valuable for those who do not find adequate relief from antihistamines or other over-the-counter remedies (108). Nasal corticosteroids can be applied alongside other allergy medications, such as antihistamines, to provide comprehensive symptom relief. The combination of therapies addresses multiple aspects of allergic reactions, enhancing overall effectiveness (110). Allergic rhinitis, if left untreated, can lead to complications like sinusitis and ear infections. Nasal corticosteroids not only alleviate symptoms and additionally contributes to preventing these secondary issues, contributing to better long-term health outcomes (110).

### 6.6 The importance of early food introduction to infants in mitigating the chance of allergies

The prevalence of childhood allergies has been steadily rising over the years, posing significant health concerns for infants and their families (111). In recent decades, researchers and healthcare professionals have explored various strategies to mitigate the risk of allergies in children. The concept of early food introduction has gained prominence as a potential approach to reduce the likelihood of developing allergies (111). Childhood allergies have emerged as a global public health challenge, impacting the well-being of millions of infants and their families. Early food introduction, the practice of introducing potentially allergenic foods to infants before six months of age, has gained attention as a preventive strategy for reducing the risk of allergies (111). The “hygiene hypothesis” suggests that reduced exposure to diverse microbial stimuli in early life may impair the development of a balanced immune system, increasing the likelihood of allergies. Early food introduction, particularly to allergenic foods, has been proposed to modulate the immune response and enhance tolerance (111).

Several allergenic foods have been under consideration for early introduction, including peanuts, eggs, milk, tree nuts, fish, and wheat. Methodologically sound studies have confirmed that the early introduction of peanuts, in particular, can drastically cut down the likelihood of developing peanut allergies in high-risk infants (111). The optimal timing and methods for early food introduction are subjects of ongoing research. Several research propose that implementing allergenic foods between four and six months of age may be most effective. Additionally, the approach of regular ingestion of the allergenic food is encouraged to promote immune tolerance (112). Despite the promising findings, early food introduction must be approached with caution. It is essential to consider individual factors, including family history of allergies and the infant's health status, before embarking on this journey. Consultation with a healthcare professional or pediatric allergist is crucial to develop a personalized and safe early food introduction plan (112). Public health initiatives and guidelines have begun to incorporate early food introduction recommendations to help parents and caregivers make informed decisions. Educational programs can play a fundamental importance in disseminating evidence-based information and empowering families to navigate the process confidently (112). Safety remains a paramount concern when introducing potentially allergenic foods to infants. Parents must be vigilant for any signs of adverse reactions, and appropriate emergency plans should be in place to manage severe allergic reactions (111).

### 6.7 Aspirin desensitization: a new approach managing hypersensitivity and allergies

Aspirin, also known as acetylsalicylic acid, is a widely used medication for pain relief and fever reduction. However, some individuals develop hypersensitivity to aspirin and related nonsteroidal anti-inflammatory drugs (NSAIDs), experiencing symptoms like asthma exacerbation, nasal polyps, and chronic sinusitis (113). Desensitization with acetylsalicylic acid has emerged as an innovative approach to managing aspirin-exacerbated respiratory disease (AERD) and other aspirin-related allergies (113). Aspirin hypersensitivity, specifically AERD, is a unique allergic condition characterized by an overreaction of the immune system to aspirin and other NSAIDs. The condition presents as a triad of asthma, nasal polyps, and sensitivity to these medications. For patients with AERD, conventional use of aspirin or NSAIDs can trigger severe and potentially life-threatening allergic reactions (113). Desensitization with acetylsalicylic acid involves a carefully administered protocol to gradually introduce aspirin to the patient in increasing doses. The process aims to retrain the immune system's response to aspirin, ultimately reducing hypersensitivity and allowing patients to tolerate the medication safely (113). During desensitization, aspirin is administered in minute, incremental doses, starting well below the threshold that would typically trigger an allergic reaction. As the dosage is gradually escalated over span of da, the immune system adapts, developing tolerance to aspirin without triggering an allergic response (114). Numerous studies have demonstrated the effectiveness of aspirin desensitization in managing AERD and aspirin-related allergies. Patients undergoing desensitization have shown improved symptom control, reduced exacerbations, and increased tolerance to aspirin and NSAIDs (114). Desensitization protocols are tailored to each patient's specific needs and medical history. The process is typically conducted in a controlled medical setting, such as a hospital or specialized clinic, with close monitoring by experienced healthcare professionals to ensure patient safety. Beyond AERD, researchers are exploring the potential of desensitization with acetylsalicylic acid in managing other aspirin-related conditions, such as chronic urticaria and idiopathic anaphylaxis (114). These investigations indicate the broad applicability of desensitization in managing diverse allergic presentations. Desensitization with acetylsalicylic acid offers patients significant benefits, including improved quality of life and enhanced medication options for pain management and other ailments. Patients who previously couldn't obtain aspirin or NSAIDs due to allergies find newfound relief and convenience through desensitization (114). While desensitization has proven effective, careful patient selection and medical supervision are crucial to minimize the risk of adverse reactions. As the understanding of aspirin hypersensitivity and desensitization continues to evolve, ongoing research and collaboration between allergists and immunologists will further optimize treatment protocols and patient outcomes (114).

### 6.8 Rapid drug desensitization in allergic treatment; a breakthrough in allergy management

Drug allergies pose significant challenges in medical practice, often limiting treatment options and patient outcomes. Drug allergies can result in life-threatening reactions and hinder essential medical treatments (115). Rapid drug desensitization, also known as drug provocation or induction of drug tolerance, offers an innovative solution to enable patients with allergies to receive vital medications (115). Rapid drug desensitization is a controlled and monitored procedure that exposes the patient to increasing doses of the allergenic drug over a short period. The process aims to induce temporary tolerance to the drug, allowing for safe administration without triggering allergic reactions (115). Rapid drug desensitization has shown efficacy in managing allergies to a broad spectrum of medications, including antibiotics, chemotherapeutic agents, and monoclonal antibodies. Patients who formerly barred from accessing these medications due to allergic reactions can now benefit from this innovative therapy (115).

The success of rapid drug desensitization relies on meticulous patient selection and personalized treatment protocols. Careful consideration of the patient's medical history, allergy severity, and the importance of the required medication is essential to optimize outcomes (116). While rapid drug desensitization is generally safe and effective, the procedure must be conducted under the supervision of experienced allergists or immunologists (116). Constant monitoring and prompt intervention in case of adverse reactions are critical to ensuring patient safety. Researchers are continually exploring new applications of rapid drug desensitization. Investigations are underway to evaluate its potential in managing allergies to other drugs and expand its utility in diverse clinical settings (117). Rapid drug desensitization has the potential to revolutionize allergy management by offering patients access to essential medications previously inaccessible because of allergies. This breakthrough therapy has the potential to improve patient outcomes and enhance quality of life significantly (117). To ensure the widespread adoption and success of rapid drug desensitization, collaboration between allergists, immunologists, and healthcare professionals is essential. Ongoing research and advancements in the field will further refine protocols and broaden the scope of this innovative therapy (117).

## 7 Discussion

The intricate relationship between pollution and allergies poses a significant and urgent public health concern with global implications. The evidence linking environmental pollutants, particularly air pollution, to allergic conditions like asthma and rhinitis is compelling. Both outdoor and indoor sources contribute to the exacerbation of allergies, affecting vulnerable populations and straining healthcare systems. Climate change, driven by escalating pollution, further complicates the scenario, intensifying seasonal allergies and increasing the demand for medical resources. The impact of pollution extends beyond human health, affecting wildlife and ecosystems, emphasizing the need for a holistic approach. Mitigating pollution's impact on allergies requires collaborative efforts from governments, industries, communities, and individuals. Stringent regulations, emission standards enforcement, and promotion of clean energy are essential governmental actions. Public awareness campaigns play a vital role in educating communities, while individual actions such as adopting sustainable practices and reducing reliance on fossil fuels contribute significantly. Improving indoor air quality is equally crucial, involving measures like proper ventilation and air purification. Recognizing the complexity of these challenges, collective action is imperative to address the root causes of pollution and their implications for both human health and the environment. By implementing effective pollution control measures and promoting sustainable practices, we can create a healthier and more sustainable future for generations to come, fostering a harmonious coexistence between humanity and nature.

## References

[1] Glencross DA Ho T-R Camiña N Hawrylowicz CM Pfeffer PE. Air pollution and its effects on the immune system. Free Radic Biol Med. (2020) 151:56–68. 10.1016/j.freeradbiomed.2020.01.179 32007522

[2] Kumar R Nagar JK Raj N Kumar P Kushwah AS Meena M Impact of domestic air pollution from cooking fuel on respiratory allergies in children in India. Asian Pac J Allergy Immunol. (2008) 26:213–22. 19317340

[3] Tiotiu AI Novakova P Nedeva D Chong-Neto HJ Novakova S Steiropoulos P Impact of air pollution on asthma outcomes. Int J Environ Res Public Health. (2020) 17:6212. 10.3390/ijerph17176212 32867076 PMC7503605

[4] Tran VV Park D Lee Y-C. Indoor air pollution, related human diseases, and recent trends in the control and improvement of indoor air quality. Int J Environ Res Public Health. (2020) 17:2927. 10.3390/ijerph17082927 32340311 PMC7215772

[5] Singh AB Kumar P. Climate change and allergic diseases: an overview. Frontiers in Allergy. (2022) 3:964987. 10.3389/falgy.2022.964987 36310569 PMC9606573

[6] Sigmund G Ågerstrand M Antonelli A Backhaus T Brodin T Diamond ML Addressing chemical pollution in biodiversity research. Glob Chang Biol. (2023) 29:3240–55. 10.1111/gcb.16689 36943240

[7] Abubakar IR Maniruzzaman KM Dano UL AlShihri FS AlShammari MS Ahmed SMS Environmental sustainability impacts of solid waste management practices in the global south. Int J Environ Res Public Health. (2022) 19:12717. 10.3390/ijerph191912717 36232017 PMC9566108

[8] Kousar S Afzal M Ahmed F Bojnec Š. Environmental awareness and air quality: the mediating role of environmental protective behaviors. Sustainability. (2022) 14:3138. 10.3390/su14063138

[9] Nwokolo SC Meyer EL Ahia CC. Credible pathways to catching up with climate goals in Nigeria. Climate. (2023) 11:196. 10.3390/cli11090196

[10] Manisalidis I Stavropoulou E Stavropoulos A Bezirtzoglou E. Environmental and health impacts of air pollution: a review. Front Public Health. (2020) 8:14. 10.3389/fpubh.2020.00014 32154200 PMC7044178

[11] Hariram NP Mekha KB Suganthan V Sudhakar K. Sustainalism: an integrated socio-economic-environmental model to address sustainable development and sustainability. Sustainability. (2023) 15:10682. 10.3390/su151310682

[12] Sun Z Zhu D. Exposure to outdoor air pollution and its human health outcomes: a scoping review. PLoS One. (2019) 14:e0216550. 10.1371/journal.pone.0216550 31095592 PMC6522200

[13] Takano H Inoue K. Environmental pollution and allergies. J Toxicol Pathol. (2017) 30:193–9. 10.1293/tox.2017-0028 28798526 PMC5545671

[14] D’Amato G Liccardi G D’Amato M Cazzola M. Outdoor air pollution, climatic changes and allergic bronchial asthma. Eur Respir J. (2002) 20:763–76. 10.1183/09031936.02.00401402 12358357

[15] Oh J-W. Pollen allergy in a changing planetary environment. Allergy Asthma Immunol Res. (2022) 14:168. 10.4168/aair.2022.14.2.168 35255535 PMC8914612

[16] Luschkova D Traidl-Hoffmann C Ludwig A. Climate change and allergies. Allergo J Int. (2022) 31:114–20. 10.1007/s40629-022-00212-x 35693463 PMC9174914

[17] Kallawicha K Chuang Y-C Lung S-CC Han B-C Ting Y-F Chao HJ. Exposure to ambient bioaerosols is associated with allergic skin diseases in greater Taipei residents. Environ Pollut. (2016) 216:845–50. 10.1016/j.envpol.2016.06.057 27389548

[18] Bonlokke JH Bang B Aasmoe L Rahman AMA Syron LN Andersson E Exposures and health effects of bioaerosols in seafood processing workers—a position statement. J Agromedicine. (2019) 24:441–8. 10.1080/1059924X.2019.1646685 31453763 PMC9048166

[19] Molina E Benedé S. Is there evidence of health risks from exposure to micro- and nanoplastics in foods? Front Nutr. (2022) 9:910094. 10.3389/fnut.2022.910094 35836585 PMC9274238

[20] Amato-Lourenço LF dos Santos Galvão L de Weger LA Hiemstra PS Vijver MG Mauad T. An emerging class of air pollutants: potential effects of microplastics to respiratory human health? Sci Total Environ. (2020) 749:141676. 10.1016/j.scitotenv.2020.141676 32827829 PMC7424328

[21] Breton CV Salam MT Wang X Byun H-M Siegmund KD Gilliland FD. Particulate matter, DNA methylation in nitric oxide synthase, and childhood respiratory disease. Environ Health Perspect. (2012) 120:1320–6. 10.1289/ehp.1104439 22591701 PMC3440108

[22] McLean MW Williamson FB. X–Carrageenase from pseudomonas carrageenovora. Eur J Biochem. (1979) 93(3):553–8. 10.1111/j.1432-1033.1979.tb12854.x 33808

[23] Kim J Oh J Sung GH. MAP kinase Hog1 regulates metabolic changes induced by hyperosmotic stress. Front Microbiol. (2016) 7:732. 10.3389/fmicb.2016.00732 27242748 PMC4870262

[24] Wang Y Xu Z Bach SJ McAllister TA. Effects of phlorotannins from Ascophyllum nodosum (brown seaweed) on in vitro ruminal digestion of mixed forage or barley grain. Anim Feed Sci Technol. (2008) 145(1–4):375–95. 10.1016/j.anifeedsci.2007.03.013

[25] Nurmatov UB Rhatigan E Simons FE Sheikh A. H1-antihistamines for primary mast cell activation syndromes: a systematic review. Allergy. (2015) 70(9):1052–61. 10.1111/all.12672 26095756

[26] Wu H Uchimura K Donnelly EL Kirita Y Morris SA Humphreys BD. Comparative analysis and refinement of human PSC-derived kidney organoid differentiation with single-cell transcriptomics. Cell Stem Cell. (2018) 23(6):869–81.e8. 10.1016/j.stem.2018.10.010 30449713 PMC6324730

[27] Khaniabadi YO Goudarzi G Daryanoosh SM Borgini A Tittarelli A De Marco A. Exposure to PM10, NO2, and O3 and impacts on human health. Environ Sci Pollut Res Int. (2017) 24:2781–9. 10.1007/s11356-016-8038-6 27837472

[28] Zhou L Liu G Shen M Hu R Sun M Liu Y. Characteristics and health risk assessment of heavy metals in indoor dust from different functional areas in Hefei, China. Environ Pollut. (2019) 251:839–49. 10.1016/j.envpol.2019.05.058 31125814

[29] Akdis CA Blaser K. Histamine in the immune regulation of allergic inflammation. J Allergy Clin Immunol. (2003) 112:15–22. 10.1067/mai.2003.1585 12847474

[30] Shaker MS Wallace DV Golden DBK Oppenheimer J Bernstein JA Campbell RL Anaphylaxis—a 2020 practice parameter update, systematic review, and grading of recommendations, assessment, development and evaluation (GRADE) analysis. J Allergy Clin Immunol. (2020) 145:1082–123. 10.1016/j.jaci.2020.01.017 32001253

[31] Matsubara T Ishikawa F Inuo C Fujita M Tsukahara A Koyama T Allergenicity of partially hydrolyzed whey and casein formulas evaluated by ImmunoCAP inhibition assay and basophil activation test. Front Allergy. (2023) 4:1207924. 10.3389/falgy.2023.1207924 37546176 PMC10403286

[32] Lin Y-T Wu C-T Huang J-L Cheng J-H Yeh K-W. Correlation of ovalbumin of egg white components with allergic diseases in children. J Microbiol Immunol Infect. (2016) 49:112–8. 10.1016/j.jmii.2014.01.002 24662019

[33] Koppelman S Smits M Tomassen M de Jong G Baumert J Taylor S Release of major peanut allergens from their matrix under various pH and simulated saliva conditions—ara h2 and ara h6 are readily bio-accessible. Nutrients. (2018) 10:1281. 10.3390/nu10091281 30208580 PMC6165493

[34] Johnson J Malinovschi A Alving K Lidholm J Borres MP Nordvall L. Ten-year review reveals changing trends and severity of allergic reactions to nuts and other foods. Acta Paediatr. (2014) 103:862–7. 10.1111/apa.12687 24825328

[35] Reese I Schäfer C Ballmer-Weber B Beyer K Dölle-Bierke S van Dullemen S Vegan diets from an allergy point of view—position paper of the DGAKI working group on food allergy. Allergol Select. (2023) 7:57–83. 10.5414/ALX02400E 37056444 PMC10088878

[36] Inomata N Miyakawa M Aihara M. High prevalence of sensitization to gibberellin-regulated protein (peamaclein) in fruit allergies with negative immunoglobulin E reactivity to Bet v 1 homologs and profilin: clinical pattern, causative fruits and cofactor effect of gibberellin-regulated protein allergy. J Dermatol. (2017) 44:735–41. 10.1111/1346-8138.13795 28326616

[37] Loh W Tang M. The epidemiology of food allergy in the global context. Int J Environ Res Public Health. (2018) 15:2043. 10.3390/ijerph15092043 30231558 PMC6163515

[38] Prescott S Allen KJ. Food allergy: riding the second wave of the allergy epidemic. Pediatr Allergy Immunol. (2011) 22:155–60. 10.1111/j.1399-3038.2011.01145.x 21332796

[39] Sampath V Abrams EM Adlou B Akdis C Akdis M Brough HA Food allergy across the globe. J Allergy Clin Immunol. (2021) 148:1347–64. 10.1016/j.jaci.2021.10.018 34872649

[40] Warren C Nimmagadda SR Gupta R Levin M. The epidemiology of food allergy in adults. Ann Allergy Asthma Immunol. (2023) 130:276–87. 10.1016/j.anai.2022.11.026 36509408

[41] De Martinis M Sirufo MM Suppa M Ginaldi L. New perspectives in food allergy. Int J Mol Sci. (2020) 21:1474. 10.3390/ijms21041474 32098244 PMC7073187

[42] Yu W Freeland DMH Nadeau KC. Food allergy: immune mechanisms, diagnosis and immunotherapy. Nat Rev Immunol. (2016) 16:751–65. 10.1038/nri.2016.111 27795547 PMC5123910

[43] Costa C Coimbra A Vítor A Aguiar R Ferreira AL Todo-Bom A. Food allergy—from food avoidance to active treatment. Scand J Immunol. (2020) 91(1):e12824. 10.1111/sji.12824 31486118

[44] Ito K Sjölander S Sato S Movérare R Tanaka A Söderström L Ige to Gly m 5 and Gly m 6 is associated with severe allergic reactions to soybean in Japanese children. J Allergy Clin Immunol. (2011) 128:673–5. 10.1016/j.jaci.2011.04.025 21555150

[45] Hassan AKG Venkatesh YP. An overview of fruit allergy and the causative allergens. Eur Ann Allergy Clin Immunol. (2015) 47:180–7. 26549334

[46] Sicherer SH Sampson HA. Food allergy: a review and update on epidemiology, pathogenesis, diagnosis, prevention, and management. J Allergy Clin Immunol. (2018) 141:41–58. 10.1016/j.jaci.2017.11.003 29157945

[47] Tomei L Muraro A Giovannini M Barni S Liccioli G Paladini E Hidden and rare food allergens in pediatric age. Nutrients. (2023) 15:1386. 10.3390/nu15061386 36986115 PMC10058883

[48] Dona A Arvanitoyannis IS. Health risks of genetically modified foods. Crit Rev Food Sci Nutr. (2009) 49:164–75. 10.1080/10408390701855993 18989835

[49] Lee T Ho H Leung T. Genetically modified foods and allergy. Hong Kong Med J. (2017) 23(3):291–5. 10.12809/hkmj166189 28473652

[50] Coimbra L Costa IM Evangelista JG Figueiredo A. Food allergens in oral care products. Sci Rep. (2023) 13:6684. 10.1038/s41598-023-33125-y 37095111 PMC10126110

[51] Agar N Freeman S. Cheilitis caused by contact allergy to cocamidopropyl betaine in ‘2-in-1 toothpaste and mouthwash’. Australas J Dermatol. (2005) 46:15–7. 10.1111/j.1440-0960.2005.00129.x 15670171

[52] Francalanci S Sertoli A Giorgini S Pigatto P Santucci B Valsecchi R. Multicentre study of allergic contact cheilitis from toothpastes. Contact Dermatitis. (2000) 43:216–22. 10.1034/j.1600-0536.2000.043004216.x 11011921

[53] Wise SK Damask C Roland LT Ebert C Levy JM Lin S International consensus statement on allergy and rhinology: allergic rhinitis—2023. Int Forum Allergy Rhinol. (2023) 13:293–859. 10.1002/alr.23090 36878860

[54] Kucuksezer UC Ozdemir C Cevhertas L Ogulur I Akdis M Akdis CA. Mechanisms of allergen-specific immunotherapy and allergen tolerance. Allergol Int. (2020) 69:549–60. 10.1016/j.alit.2020.08.002 32900655

[55] Shamji MH Sharif H Layhadi JA Zhu R Kishore U Renz H. Diverse immune mechanisms of allergen immunotherapy for allergic rhinitis with and without asthma. J Allergy Clin Immunol. (2022) 149:791–801. 10.1016/j.jaci.2022.01.016 35093483

[56] Racanelli AC Kikkers SA Choi AMK Cloonan SM. Autophagy and inflammation in chronic respiratory disease. Autophagy. (2018) 14:221–32. 10.1080/15548627.2017.1389823 29130366 PMC5902194

[57] Dunlop J Matsui E Sharma HP. Allergic rhinitis. Immunol Allergy Clin North Am. (2016) 36:367–77. 10.1016/j.iac.2015.12.012 27083109

[58] Stefanovic N Irvine AD Flohr C. The role of the environment and exposome in atopic dermatitis. Curr Treat Options Allergy. (2021) 8:222–41. 10.1007/s40521-021-00289-9 34055570 PMC8139547

[59] Simon D. Recent advances in clinical allergy and immunology 2019. Int Arch Allergy Immunol. (2019) 180:291–305. 10.1159/000504364 31694018

[60] Li Y Rui X Ma B Jiang F Chen J. Early-life environmental factors, IFN-γ methylation patterns, and childhood allergic rhinitis. Int Arch Allergy Immunol. (2019) 178:323–32. 10.1159/000495304 30612129

[61] Kojima R Shinohara R Kushima M Horiuchi S Otawa S Miyake K Effect of birth season on allergic rhinitis and cedar pollinosis considering allergen and vitamin D exposure: the Japan environment and Children’s study (JECS). Allergol Int. (2023) 72:411–7. 10.1016/j.alit.2023.01.003 36725444

[62] Desalu OO Adeoti AO Ojuawo OB Aladesanmi AO Oguntoye MS Afolayan OJ Urban–rural differences in the epidemiology of asthma and allergies in Nigeria: a population-based study. J Asthma Allergy. (2021) 14:1389–97. 10.2147/JAA.S333133 34866916 PMC8637762

[63] Lang JE. The impact of exercise on asthma. Curr Opin Allergy Clin Immunol. (2019) 19:118–25. 10.1097/ACI.0000000000000510 30601152

[64] Cazzola M Polosa R. Anti-TNF-α and Th1 cytokine-directed therapies for the treatment of asthma. Curr Opin Allergy Clin Immunol. (2006) 6:43–50. 10.1097/01.all.0000199798.10047.74 16505611

[65] Asher MI. Urbanisation, asthma and allergies. Thorax. (2011) 66:1025–6. 10.1136/thoraxjnl-2011-201019 21948729 PMC3221320

[66] Eguiluz-Gracia I Mathioudakis AG Bartel S Vijverberg SJH Fuertes E Comberiati P The need for clean air: the way air pollution and climate change affect allergic rhinitis and asthma. Allergy. (2020) 75:2170–84. 10.1111/all.14177 31916265

[67] Chatkin J Correa L Santos U. External environmental pollution as a risk factor for asthma. Clin Rev Allergy Immunol. (2022) 62:72–89. 10.1007/s12016-020-08830-5 33433826 PMC7801569

[68] Riggs DW Zafar N Krishnasamy S Yeager R Rai SN Bhatnagar A Exposure to airborne fine particulate matter is associated with impaired endothelial function and biomarkers of oxidative stress and inflammation. Environ Res. (2020) 180:108890. 10.1016/j.envres.2019.108890 31718786 PMC6899204

[69] Keller JP Larson TV Austin E Barr RG Sheppard L Vedal S Pollutant composition modification of the effect of air pollution on progression of coronary artery calcium. Environ Epidemiol. (2018) 2:e024. 10.1097/EE9.0000000000000024 30854505 PMC6402342

[70] Sun S Stewart JD Eliot MN Yanosky JD Liao D Tinker LF Short-term exposure to air pollution and incidence of stroke in the Women’s health initiative. Environ Int. (2019) 132:105065. 10.1016/j.envint.2019.105065 31382185 PMC6754774

[71] Wang M Aaron CP Madrigano J Hoffman EA Angelini E Yang J Association between long-term exposure to ambient air pollution and change in quantitatively assessed emphysema and lung function. JAMA. (2019) 322:546. 10.1001/jama.2019.10255 31408135 PMC6692674

[72] Dadvand P Parker J Bell ML Bonzini M Brauer M Darrow LA Maternal exposure to particulate air pollution and term birth weight: a multi-country evaluation of effect and heterogeneity. Environ Health Perspect. (2013) 121:267–373. 10.1289/ehp.1205575 23384584 PMC3621183

[73] Shi L Wu X Danesh Yazdi M Braun D Abu Awad Y Wei Y Long-term effects of PM2·5 on neurological disorders in the American medicare population: a longitudinal cohort study. Lancet Planet Health. (2020) 4:e557–65. 10.1016/S2542-5196(20)30227-8 33091388 PMC7720425

[74] Cheng I Tseng C Wu J Yang J Conroy SM Shariff-Marco S Association between ambient air pollution and breast cancer risk: the multiethnic cohort study. Int J Cancer. (2020) 146:699–711. 10.1002/ijc.32308 30924138 PMC6765455

[75] Niehoff NM Gammon MD Keil AP Nichols HB Engel LS Sandler DP Airborne mammary carcinogens and breast cancer risk in the sister study. Environ Int. (2019) 130:104897. 10.1016/j.envint.2019.06.007 31226564 PMC6679994

[76] Smith MT Jones RM Smith AH. Benzene exposure and risk of non-hodgkin lymphoma. Cancer Epidemiol Biomarkers Prev. (2007) 16:385–91. 10.1158/1055-9965.EPI-06-1057 17337645

[77] Lin C-K Lin R-T Chen T Zigler C Wei Y Christiani DC. A global perspective on coal-fired power plants and burden of lung cancer. Environ Health. (2019) 18:9. 10.1186/s12940-019-0448-8 30691464 PMC6350330

[78] Frew AJ. Allergen immunotherapy. J Allergy Clin Immunol. (2010) 125:S306–13. 10.1016/j.jaci.2009.10.064 20176266

[79] Şahin E Ali Bafaqeeh S Güven SG Çetinkaya EA Muluk NB Coşkun ZO Mechanism of action of allergen immunotherapy. Am J Rhinol Allergy. (2016) 30:S1–3. 10.2500/ajra.2016.30.4367 27658024

[80] Dhami S Kakourou A Asamoah F Agache I Lau S Jutel M Allergen immunotherapy for allergic asthma: a systematic review and meta-analysis. Allergy. (2017) 72:1825–48. 10.1111/all.13208 28543086

[81] Bumbacea RS Boustani R Panaitescu C Haidar L Buzan M-R Bumbacea D Mechanisms of allergen immunotherapy supporting its disease-modifying effect. Immunotherapy. (2022) 14:627–38. 10.2217/imt-2021-0325 35416072

[82] James C Bernstein DI. Allergen immunotherapy: an updated review of safety. Curr Opin Allergy Clin Immunol. (2017) 17:55–9. 10.1097/ACI.0000000000000335 27906697 PMC5644500

[83] Schworer SA Kim EH. Sublingual immunotherapy for food allergy and its future directions. Immunotherapy. (2020) 12:921–31. 10.2217/imt-2020-0123 32611211 PMC7421796

[84] Hesse L Oude Elberink JNG van Oosterhout AJM Nawijn MC. Allergen immunotherapy for allergic airway diseases: use lessons from the past to design a brighter future. Pharmacol Ther. (2022) 237:108115. 10.1016/j.pharmthera.2022.108115 35063570

[85] Durham SR Shamji MH. Allergen immunotherapy: past, present and future. Nat Rev Immunol. (2023) 23:317–28. 10.1038/s41577-022-00786-1 36253555 PMC9575636

[86] Senti G Kündig TM. Intralymphatic immunotherapy. World Allergy Organ J. (2015) 8:9. 10.1186/s40413-014-0047-7 25780493 PMC4352255

[87] Senti G Johansen P Kündig TM. Intralymphatic immunotherapy: from the rationale to human applications. Curr Top Microbiol Immunol. (2011) 352:71–84. 10.1007/82_2011_133 21725898

[88] Senti G Freiburghaus AU Larenas-Linnemann D Hoffmann HJ Patterson AM Klimek L Intralymphatic immunotherapy: update and unmet needs. Int Arch Allergy Immunol. (2019) 178:141–9. 10.1159/000493647 30391954

[89] Sugiura S Kitamura K Makino A Matsui T Furuta T Takasato Y Slow low-dose oral immunotherapy: threshold and immunological change. Allergol Int. (2020) 69:601–9. 10.1016/j.alit.2020.03.008 32444309

[90] Yanagida N Okada Y Sato S Ebisawa M. New approach for food allergy management using low-dose oral food challenges and low-dose oral immunotherapies. Allergol Int. (2016) 65:135–40. 10.1016/j.alit.2015.10.010 26774524

[91] Barshow SM Kulis MD Burks AW Kim EH. Mechanisms of oral immunotherapy. Clin Exp Allergy. (2021) 51:527–35. 10.1111/cea.13824 33417257 PMC9362513

[92] Kawauchi H Yanai K Wang D-Y Itahashi K Okubo K. Antihistamines for allergic rhinitis treatment from the viewpoint of nonsedative properties. Int J Mol Sci. (2019) 20:213. 10.3390/ijms20010213 30626077 PMC6337346

[93] Kuna P Jurkiewicz D Czarnecka-Operacz MM Pawliczak R Woroń J Moniuszko M The role and choice criteria of antihistamines in allergy management—expert opinion. Adv Dermatol Allergol. (2016) 6:397–410. 10.5114/pdia.2016.63942 28035215 PMC5183790

[94] Fein MN Fischer DA O’Keefe AW Sussman GL. CSACI Position statement: newer generation H1-antihistamines are safer than first-generation H1-antihistamines and should be the first-line antihistamines for the treatment of allergic rhinitis and urticaria. Allergy Asthma Clin Immunol. (2019) 15:61. 10.1186/s13223-019-0375-9 31582993 PMC6771107

[95] Church MK Maurer M Simons FER Bindslev-Jensen C Van Cauwenberge P Bousquet J Risk of first-generation H_1_ -antihistamines: a GA^2^ LEN position paper. Allergy. (2010) 65:459–66. 10.1111/j.1398-9995.2009.02325.x 20146728

[96] Zhang T Finn DF Barlow JW Walsh JJ. Mast cell stabilisers. Eur J Pharmacol. (2016) 778:158–68. 10.1016/j.ejphar.2015.05.071 26130122

[97] Can I Samim E. Mast cell stabilizers in the treatment of allergic rhinitis. Antiinflamm Antiallergy Agents Med Chem. (2008) 7:9–15. 10.2174/187152308783769177

[98] Shin H-Y Kim J-S An N-H Park R-K Kim H-M. Effect of disodium cromoglycate on mast cell-mediated immediate-type allergic reactions. Life Sci. (2004) 74:2877–87. 10.1016/j.lfs.2003.10.026 15050425

[99] Shaw RJ Kay AB. Nedocromil, a mucosal and connective tissue mast cell stabilizer, inhibits exercise-induced asthma. Br J Dis Chest. (1985) 79:385–9. 10.1016/0007-0971(85)90073-7 2996580

[100] Rosenwasser LJ O’Brien T Weyne J. Mast cell stabilization and anti-histamine effects of olopatadine ophthalmic solution: a review of pre-clinical and clinical research. Curr Med Res Opin. (2005) 21:1377–87. 10.1185/030079905X56547 16197656

[101] Mishra GP Tamboli V Jwala J Mitra AK. Recent patents and emerging therapeutics in the treatment of allergic conjunctivitis. Recent Pat Inflamm Allergy Drug Discov. (2011) 5:26–36. 10.2174/187221311794474883 21171952 PMC3164156

[102] Zuraw BL Busse PJ White M Jacobs J Lumry W Baker J Nanofiltered C1 inhibitor concentrate for treatment of hereditary angioedema. N Engl J Med. (2010) 363:513–22. 10.1056/NEJMoa0805538 20818886

[103] Davis AE. New treatments addressing the pathophysiology of hereditary angioedema. Clin Mol Allergy. (2008) 6:2. 10.1186/1476-7961-6-2 18410689 PMC2374835

[104] Riedl MA Hurewitz DS Levy R Busse PJ Fitts D Kalfus I. Nanofiltered C1 esterase inhibitor (human) for the treatment of acute attacks of hereditary angioedema: an open-label trial. Ann Allergy Asthma Immunol. (2012) 108:49–53. 10.1016/j.anai.2011.10.017 22192966

[105] Li HH. Self-administered C1 esterase inhibitor concentrates for the management of hereditary angioedema: usability and patient acceptance. Patient Prefer Adherence. (2016) 10:1727–37. 10.2147/PPA.S86379 27660422 PMC5019432

[106] Zuraw BL Kalfus I. Safety and efficacy of prophylactic nanofiltered C1-inhibitor in hereditary angioedema. Am J Med. (2012) 125:938.e1–e7. 10.1016/j.amjmed.2012.02.020 22800873

[107] Tourangeau LM Zuraw BL. The new era of C1-esterase inhibitor deficiency therapy. Curr Allergy Asthma Rep. (2011) 11:345–51. 10.1007/s11882-011-0213-8 21833753

[108] Watson W Becker A Simons F. Treatment of allergic rhinitis with intranasal corticosteroids in patients with mild asthma: effect on lower airway responsiveness. J Allergy Clin Immunol. (1993) 91:97–101. 10.1016/0091-6749(93)90301-U 8423275

[109] Okano M. Mechanisms and clinical implications of glucocorticosteroids in the treatment of allergic rhinitis. Clin Exp Immunol. (2009) 158:164–73. 10.1111/j.1365-2249.2009.04010.x 19737138 PMC2768806

[110] LaForce C. Use of nasal steroids in managing allergic rhinitis. J Allergy Clin Immunol. (1999) 103:S388–94. 10.1016/S0091-6749(99)70218-6 10069899

[111] Comberiati P Costagliola G D’Elios S Peroni D. Prevention of food allergy: the significance of early Introduction. Medicina. (2019) 55:323. 10.3390/medicina55070323 31261990 PMC6681183

[112] Sansotta N Piacentini GL Mazzei F Minniti F Boner AL Peroni DG. Timing of introduction of solid food and risk of allergic disease development: understanding the evidence. Allergol Immunopathol. (2013) 41:337–45. 10.1016/j.aller.2012.08.012 23287585

[113] Breslow RG Caiado J Castells MC. Acetylsalicylic acid and montelukast block mast cell mediator–related symptoms during rapid desensitization. Ann Allergy Asthma Immunol. (2009) 102:155–60. 10.1016/S1081-1206(10)60247-5 19230468

[114] Ibrahim C Singh K Tsai G Huang D Mazza J Rotenberg B A retrospective study of the clinical benefit from acetylsalicylic acid desensitization in patients with nasal polyposis and asthma. Allergy Asthma Clin Immunol. (2014) 10:64. 10.1186/s13223-014-0064-7 25516728 PMC4267150

[115] Castells M. Rapid desensitization for hypersensitivity reactions to medications. Immunol Allergy Clin North Am. (2009) 29:585–606. 10.1016/j.iac.2009.04.012 19563999

[116] Warrington R. Drug allergy. Hum Vaccin Immunother. (2012) 8:1513–24. 10.4161/hv.21889 22922763 PMC3660773

[117] de las Vecillas Sánchez L Alenazy L Garcia-Neuer M Castells M. Drug hypersensitivity and desensitizations: mechanisms and new approaches. Int J Mol Sci. (2017) 18:1316. 10.3390/ijms18061316 28632196 PMC5486137

